# Multi-Domain Negative Capacitance Effects in Metal-Ferroelectric-Insulator-Semiconductor/Metal Stacks: A Phase-field Simulation Based Study

**DOI:** 10.1038/s41598-020-66313-1

**Published:** 2020-06-23

**Authors:** Atanu K. Saha, Sumeet K. Gupta

**Affiliations:** School of Electrical and Computer Engineering, Purdue University, West Lafayette, IN 47907 USA

**Keywords:** Electrical and electronic engineering, Electronic devices

## Abstract

We analyze the ferroelectric domain-wall induced negative capacitance (NC) effect in Metal-FE-Insulator-Metal (MFIM) and Metal-FE-Insulator-Semiconductor (MFIS) stacks through phase-field simulations by self-consistently solving time-dependent Ginzburg Landau equation, Poisson’s equation and semiconductor charge equations. Considering Hf_0.5_Zr_0.5_O_2_ as the ferroelectric material, we study 180° ferroelectric domain formation in MFIM and MFIS stacks and their polarization switching characteristics. Our analysis signifies that the applied voltage-induced polarization switching via soft domain-wall displacement exhibits non-hysteretic characteristics. In addition, the change in domain-wall energy, due to domain-wall displacement, exhibits a long-range interaction and thus, leads to a non-homogeneous effective local negative permittivity in the ferroelectric. Such effects yield an average negative effective permittivity that further provides an enhanced charge response in the MFIM stack, compared to Metal-Insulator-Metal. Furthermore, we show that the domain-wall induced negative effective permittivity is not an intrinsic property of the ferroelectric material and therefore, is dependent on its thickness, the gradient energy coefficient and the in-plane permittivity of the underlying insulator. Similar to the MFIM stack, MFIS stack also exhibits an enhanced charge/capacitance response compared to Metal-Oxide-Semiconductor (MOS) capacitor. Simultaneously, the multi-domain state of the ferroelectric induces non-homogeneous potential in the underlying insulator and semiconductor layer. At low applied voltages, such non-homogeneity leads to the co-existence of electrons and holes in an undoped semiconductor. In addition, we show that with the ferroelectric layer being in the 180° multi-domain state, the minimum potential at the ferroelectric-dielectric interface and hence, the minimum surface potential in the semiconductor, does not exceed the applied voltage (in-spite of the local differential amplification and charge enhancement).

## Introduction

The negative capacitance (NC) effect in ferroelectric (FE) materials has attracted an immense attention because of its potential to overcome the fundamental limits in field-effect transistor (FET) operation^1^. In conventional Metal-Oxide-Semiconductor (MOS) FET, only a fraction of the applied gate voltage (${V}_{app}$) appears as semiconductor surface potential (Ψ) due to a voltage drop across the positive gate dielectric capacitance and therefore, *d*Ψ/$d{V}_{app} < 1$. Consequently, the attainable sub-threshold swing (SS) in MOSFET is always higher than 60 mV/decade at room temperature (300 K)^1^. Such a fundamental limit of SS, further limits the voltage scalability of MOSFET without sacrificing the drive current. To overcome such limitations, it has been proposed that an FE layer in the gate stack of FE-FET can act as an effective negative capacitor under certain conditions^1^. Most importantly, such negative capacitance effects can provide differential amplification of the interface potential which leads to *d*Ψ/$d{V}_{app} > 1$ and SS < 60 mV/decade^1^. As a result, the NC effect in FE materials can potentially enable voltage scaling without affecting the drive current of FE-FET.

**Figure 1:**
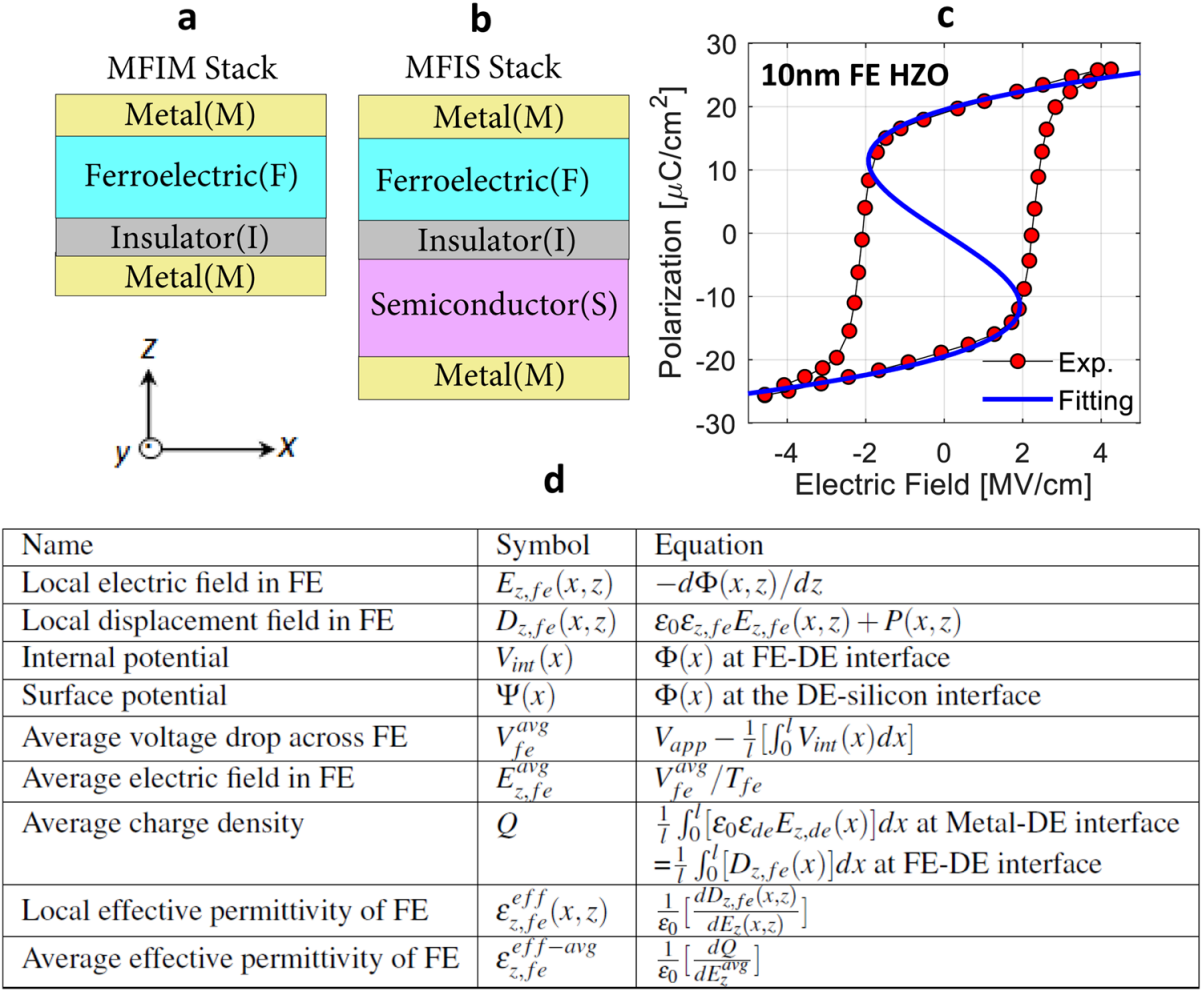
(**a**) Metal-Ferroelectric-Insulator-Metal (MFIM) and (**b**) Metal-Ferroelectric-Insulator-Semiconductor (MFIS) configurations. (**c**) Calibration of Landau’s free energy coefficients with experimental^12^ *P*-*E* characteristics of 10 nm HZO thin-film. The calibrated Landau coefficients are: $\alpha =-\,2.5\times {10}^{9}\,Vm/C$, $\beta =6.0\times {10}^{10}\,V{m}^{5}/{C}^{3}$ and $\gamma =1.5\times {10}^{11}\,V{m}^{9}/{C}^{5}$. (**d**) Tabular representation of symbols and their corresponding definitions.

According to Landau-Khalatnikov equation^1^, FE polarization ($P$) versus electric-field ($E$) characteristics exhibit an unstable negative slope (negative $dP/dE$) region as shown in Fig. 1(c). According to ref. ^1^, such an unstable region in FE can be stabilized in a heterogeneous system (i.e. FE-dielectric (DE) stack) so that a homogeneously suppressed polarization (*P* = 0) can be obtained by suppressing the depolarization energy. However, under certain conditions, it may be more natural to form multiple domains with positive and negative $P$ separated by domain-walls (DWs) to suppress the depolarization energy of the system^2^. Recently, DW motion-based $P$-switching in multi-domain (MD) FE has been identified as a possible mechanism for obtaining static effective negative capacitance in FE^2–5^. Such DW-induced NC effect has been theoretically predicted in refs. ^6–8^ showing that the soft-DW displacement can lead to an effective negative permittivity of FE in the presence of the interfacial dead layer. Further, a similar effect has been discussed and analyzed through phase-field simulations predicting a hysteresis-free NC path in FE by considering a moving DW in a metal-FE-metal capacitor^9^ and DE-FE-DE superlattice^10^. Additionally, an analytical model for DW-induced NC has been proposed for DE-FE-DE superlattice in ref. ^10^ suggesting that the effective NC path is dependent on the DE thickness (${T}_{de}$), which contrasts with the analytical model presented in ref. ^11^. Our phase-field simulations show that the soft-DW motion-based effective NC path in FE is independent of ${T}_{de}$, but it depends on the in-plane permittivity of the DE layer, which is in agreement with ref. ^11^. To further identify such inter-dependencies of FE NC behavior on the properties of the constituent FE and DE layers in such heterostructures, we extensively analyze DW-induced NC effect in MFIM (Fig. 1(a)) based on phase-field simulations (beyond what has been explored so far) and establish its dependence on FE thickness, gradient energy coefficient, and DE permittivity and thickness. Furthermore, we, for the first time, develop a self-consistent 2D phase-field simulation framework for Metal-Ferroelectric-Insulator-Semiconductor (MFIS) stack (Fig. 1(b)). Utilizing our framework, we investigate DW induced NC effect in the MFIS stack, its effect on the semiconductor potential and its dependency on key material/device parameters.

In our phase-field simulation, we solve the 2D time ($t$)-dependent Ginzburg-Landau (TDGL) equation^10^, Poisson’s equation and semiconductor charge equations, yielding self-consistent solutions for polarization ($P(x,z,t)$), potential (Φ$(x,z,t)$) and charge ($\rho (x,z,t)$), where $z$ and $x$ are the axis parallel to the thickness and length of the stack, respectively. Even though the simulation framework can capture the transient dynamics of polarization switching, in this work, we focus on analyzing the steady-state solution of $P(x,z)$ for an applied voltage. For the FE material, we consider Hf_0.5_Zr_0.5_O_2_ (HZO) and the corresponding Landau’s free energy coefficients ($\alpha$, $\beta$ and $\gamma$) are extracted from measured $P$-$E$ characteristics^12^ shown in Fig. 1(c). For the gradient energy coefficient ($g$), a range of values are considered (see Methods section) as the actual value is still unknown for HZO. We assume that the spontaneous $P$ direction in FE is along the thickness of the film ($z$-axis), which is parallel to the *c*-axis of the orthorhombic crystal phase of HZO^13,14^. For DE, we consider SiO_2_, Al_2_O_3_ and HfO_2_, and for the semiconductor, we consider undoped silicon. The details of the simulation methodologies, parameters and calibration are discussed in the ‘Methods’ section. In addition, the symbolic representations and corresponding equations of different parameters are summarized in a table shown in Fig. 1(d). Utilizing this framework, we analyze the multi-domain formation and the applied voltage-dependent $P$-switching in MFIM and MFIS stacks (shown in Fig. 1(a,b)), which we discuss in the subsequent sections.

## Multi-domain formation in MFIM stack

**Figure 2:**
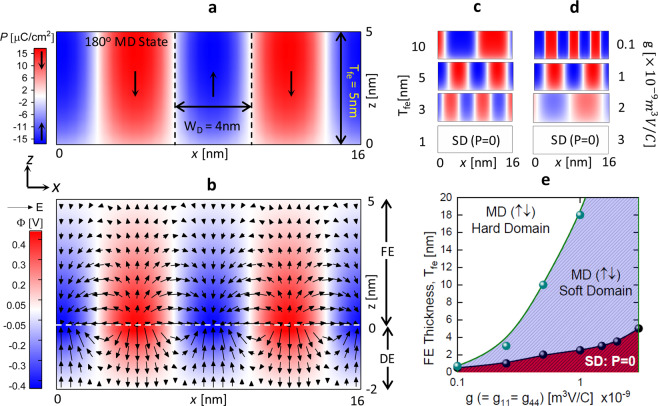
(**a**) Polarization profile of FE ($P(x,z)$) and (**b**) potential profile (Φ(*x*, *z*)) of MFIM stack at *V*_*app*_ = 0 V showing 180° domain formation in FE. Here, *T*_*fe*_ = 5 nm, *T*_*de*_ = 2 nm, *ε*_*de*_ = 10 (Al_2_O_3_). Polarization profiles of FE in MFIM for (**c**) different *T*_*fe*_ and (**d**) different *g* at *V*_*app*_ = 0 V. (**e**) *g* vs critical *T*_*fe*_ for MD (P$\uparrow \downarrow$) state with soft-DW, hard-DW and SD (P = 0) state for *ε*_*de*_ = 10. The lateral dimension (along the *x*-axis) of 100 nm is used for all simulations.

Let us start by considering an MFIM stack with an applied voltage (${V}_{app}$) of 0 V. It is well known that in MFIM stack, spontaneous polarization ($P$) appears at the FE-DE interface, which leads to a voltage drop across the DE layer^10^. As a result, an E-field appears in FE opposite to the $P$ direction, called depolarization field, which leads to an increase in the depolarization energy density. Let us define the E-field along the thickness of the FE film (z-axis) as ${E}_{z,fe}$ and the depolarization energy density as ${f}_{dep}=-\,{E}_{z,fe}\times P$. However, ${f}_{dep}$ can be suppressed with the formation of periodic 180° domains of alternating $P$-directions (P$\downarrow$ and P$\uparrow$). Simulation result considering such a scenario is shown in Fig. 2(a) for *T*_*fe*_ = 5 nm, *T*_*de*_ = 2 nm (Al_2_O_3_), $g=1\times {10}^{-9}\,{m}^{3}V/C$. Here, $\uparrow$ ($\downarrow$) sign denotes the +*z* (−*z*) direction. In this multi-domain (MD) state, the magnitude of the local ${E}_{z,fe}$ (at a particular point in the FE) is greatly reduced due to stray fields (in-plane E-field, ${E}_{x,fe}$) between P$\uparrow$ and P$\downarrow$ domains, as shown in Fig. 2(b). While this decrease in local ${E}_{z,fe}$ is larger near the domain walls (DWs) compared to inside of the domains, the suppression of average ${E}_{z,fe}$ is significant across the entire length of the stack (along the $x$-direction). The resultant decrease in ${f}_{dep}$, however, comes at the cost of DW energy density (${F}_{dw}$), which is comprised of (a) the electrostatic energy density (${f}_{x,elec}={\varepsilon }_{0}{\varepsilon }_{x,fe}\times {E}_{x,fe}^{2}$) due to stray fields, where ${\varepsilon }_{x,fe}$ is the in-plane background permittivity of FE and (b) $x$-component of gradient energy density (${f}_{x,grad}=g\times {(dP/dx)}^{2}$) due to the spatial variation in $P$ along the $x$-axis. Subsequently, we will refer to the sum of ${f}_{x,grad}$ and ${f}_{x,elec}$ over the FE region as the DW energy (${F}_{dw}=\iint \,{f}_{dw}dxdz$, where, ${f}_{dw}={f}_{x,grad}+{f}_{x,elec}$). Note that the magnitude of $P$ inside of a domain also varies along the $z$-axis exhibiting a minima at the DE interface and gradually increases in the bulk FE (away from the DE interface). This induces a bound charge density ${\rho }_{b}=-\,dP/dz$ and further suppresses the ${E}_{z,fe}$ (and hence, ${f}_{dep}$) inside of the domain. However, this additional suppression of ${E}_{z,fe}$ occurs at the cost of an increase in the $z$-component of gradient energy density (${f}_{z,grad}=g\times {(dP/dz)}^{2}$). Our simulations show that ${f}_{z,grad}$ occurs in FE both in the MD (co-existing P$\uparrow$ and P$\downarrow$) and poled (either P$\uparrow$ or P$\downarrow$) states. In the MD state, achieved by suppressing ${f}_{dep}$ at the cost of ${f}_{dw}$ and ${f}_{z,grad}$ while minimizing the overall system energy, the intricate interactions of these energy components with each other as well as the free energy (${f}_{free}$) play a key role in determining the charge response of FE in the MFIM stack and its dependence on the device/material parameters, as discussed subsequently.

Now, let us first consider the implication of FE thickness, ${T}_{fe}$ on the formation of MD state. The $P$ configurations of FE in MFIM stacks shown in Fig. 2(c), suggest that the number of domains and DWs (within a certain length) increases with the decrease in ${T}_{fe}$. As ${T}_{fe}$ decreases, ${f}_{z,grad}$ increases as a similar $P$ variation along $z$-axis (i.e. similar $P$ maxima in the bulk and minima in the interface) occurs within a lower ${T}_{fe}$. One of the possible ways to reduce ${f}_{z,grad}$ could be decreasing $P$ variation by increasing $P$ magnitude in the interface, but this would increase ${f}_{dep}$. As an alternative, an increase in the number of DWs leads to reduced domain width and thus, reduces the $P$ magnitude in the bulk region. Therefore, an increase in ${f}_{z,grad}$ due to ${T}_{fe}$ scaling is mitigated by increasing the number of DWs. In this case, suppression of ${f}_{dep}$ becomes more significant inside of a domain (as $P$ decreases in magnitude) and also on an average (as the number of DWs increases). At the same time, with decreasing ${T}_{fe}$, as the number of DWs increases, the nature of DW changes from hard to soft type (Fig. 2(c)). Here, the term hard-DW implies that the spatial variation in $P$ is localized and abrupt ($dP/dx$ is high) only near the DWs. Thus, ${f}_{x,grad}$ is non-zero in the DW and zero (or very small) within the domains. In contrast, in a soft-DW, the $P$ variation is more gradual ($dP/dx$ is low) and the effects of DW diffuses along the length-scale of a domain. That implies ${f}_{x,grad}$ becomes non-zero inside of a domain.

Similar to the effect of ${T}_{fe}$, the gradient energy coefficient ($g$) also determines the number of DWs in FE. As ${f}_{x,grad}$ is one of the components of ${f}_{dw}$, a decrease in $g$ leads to lower DW energy cost and, thus, leads to larger number of domains and DWs. Such an increase in the number of DWs density with the decrease in $g$ is shown in Fig. 2(d). Further, the nature of DW changes from hard to soft type as $g$ increases. This is because, for higher $g$, $dP/dx$ decreases to compensate for the ${f}_{x,grad}$ and thus the $P$-variation becomes more gradual and diffuses within the domain. The nature of DW (i.e. soft or hard) in MFIM for different $g$ and ${T}_{fe}$ is illustrated in Fig. 2(e) showing that the critical ${T}_{fe}$ for hard to soft DW transition decreases with a decrease in $g$. Further, if ${T}_{fe}$ is scaled below a critical value, a single domain (SD) state with homogenous $P$ = 0 stabilizes (Fig. 2(c): ${T}_{fe}$ = 1 nm), where the suppression of ${f}_{dep}$ occurs at the cost of ${f}_{free}$ rather than ${f}_{dw}$. For suppressing ${f}_{dep}$, if ${f}_{dw}$ is higher than ${f}_{free}$ then the SD state is preferred over the MD state. In addition, the critical ${T}_{fe}$, for MD to SD transition, decreases with a decrease in $g$ as shown in Fig. 2(e). As ${f}_{dw}$ decreases with $g$, a lower ${T}_{fe}$ is needed to go beyond ${f}_{free}$. Note that if $g$ is very small, the critical ${T}_{fe}$ can potentially become so small that the SD state or soft MD state may not be physically realizable. For example, if $g < 0.1\times {10}^{-9}\,{m}^{3}V/C$, the critical ${T}_{fe}$ for SD state (0.25 nm) and soft MD state (0.5 nm) is lower than or comparable to a unit cell height of HZO.

In the above discussion, the stability of SD state with *P* = 0 may or may not imply that the ferroelectricity of the FE layer will be retained and the same Landau coefficients can be used to calculate the polarization switching characteristics. Rather, one needs to calculate the ${f}_{free}$ of the ferroelectric phase at $P$ = 0 along with the paraelectric and anti-ferroelectric phases (i.e. orthorhombic, monoclinic and tetragonal phase for HZO). In such a case, the state with lowest ${f}_{free}$ will be stabilized and if the stable phase is either paraelectric or anit-ferroelectric, then the Landau coefficients for ferroelectric phase can not be used to simulate the polarization switching characteristics. However, such calculation requires first-principle simulation, which is out of the scope of our current study. Hence, we limit our analysis to the MD states.

Further, in the MD state, the nature of DW plays an important role in E-field driven DW motion. To displace the hard-DW, the applied E-field needs to be higher than a critical value called coercive field of DW motion, ${E}_{c,dw}$. Therefore, the hard-DW motion based $P$-switching is hysteretic, because, the applied E-field needs to be higher than the positive ${E}_{c,dw}$ for forward DW motion and lower than the negative ${E}_{c,dw}$ for reverse DW motion^15^. In contrast, $|{E}_{c,dw}|$ is infinitesimally small (~0) for soft-DW^15^ and hence, non-hysteretic DW motion is possible. As in this work, our focus is on analyzing the non-hysteretic NC effect, we restrict our discussion only for soft-DW motion based $P$-switching.

## MD-NC Effect in MFIM Stack

**Figure 3:**
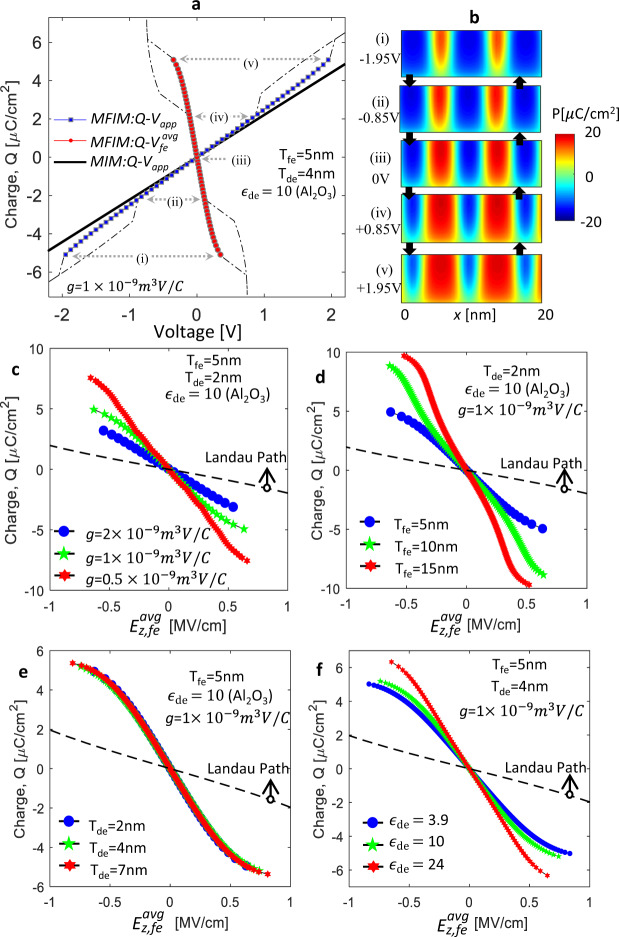
(**a**) *Q*-*V*_*app*_ characteristics of MFIM stack when FE is in MD state (blue) showing enhanced charge response of MFIM stack compared to MIM (Metal-DE-Metal black-solid) stack. The black-dashed line represents the poled state (if *V*_*app*_ > 2 V). *Q*-${V}_{fe}^{avg}$ response (red-circle). (**b**) $P(x,z)$ of FE at different ${V}_{app}$ (as marked in Fig. 3(a)). *Q*-${E}_{z,fe}^{avg}$ characteristics of FE in MFIM stack considering (**c**) different *g*, (**d**) different *T*_*fe*_, (**e**) different *T*_*de*_ and (**f**) different *ε*_*de*_. black-dashed line in (**c**–**f**) is the single domain Landau path. The lateral dimension (along the x-axis) of 100 nm is used for all simulations.

Let us begin by discussing *P*-switching in the MFIM stack with soft-DW. The simulated average charge density ($Q$) vs applied voltage (${V}_{app}$) characteristics is shown in Fig. 3(a) for ${T}_{fe}$ = 5 nm, ${T}_{de}$ = 4 nm (Al_2_O_3_), $g=1\times {10}^{-9}\,{m}^{3}V/C$. Here, $Q$ is calculated as the average displacement field at the Metal-DE interface based on following equation.

1 $$Q=\frac{1}{l}[{\int }_{0}^{l}\,{\varepsilon }_{0}{\varepsilon }_{de}\times {E}_{z,de}(x)dx]$$

where, ${E}_{z,de}$ is the z-component of E-field at Metal-DE interface and $l$ is the length of the stack. For $|{V}_{app}| < 2\,\text{V}$, a continuous $Q$-${V}_{app}$ path exists when the FE is in the MD state and $P$-switching takes place through DW motion (see Fig. 3(b)). If $|{V}_{app}|$ is increased above 2 V, MD state (*P*) switches to the poled state (either $P\uparrow$ or $P\downarrow$). Now, with decreasing $|{V}_{app}|$, MD state forms from the poled state at a lower $|{V}_{app}|$ (0.9 V) and that induces a hysteresis in the $Q$-${V}_{app}$ characteristics (discussed in the Supplementary Section). Therefore, for non-hysteretic operation, the MD state needs to be retained by limiting ${V}_{app}$. Interestingly, in the MD state, $Q$ is higher in the MFIM stack compared to the MIM (Metal-Insulator-Metal) at the same ${V}_{app}$ as shown in Fig. 3(a). This implies that the effective capacitance of the MFIM stack is higher than MIM. In a static scenario, such a phenomena is only possible if the FE layer acts as an effective negative capacitor. The $Q-{V}_{fe}^{avg}$ characteristics are shown in Fig. 3(a). In the MD state, as the potential drop across the FE layer is non-homogeneous along the x-axis (Fig. 2(b)), the average potential drop across the FE layer (${V}_{fe}^{avg}$) is calculated using the following equation.

2 $${V}_{fe}^{avg}={V}_{app}-\frac{1}{l}[{\int }_{0}^{l}\,{V}_{{int}}(x)dx]$$

Here, ${V}_{int}(x)$ is the FE-DE interface potential $Q$ is calculated by taking the average displacement field at the FE-DE interface which provides exactly same $Q$ as Eq. 1 for the same ${V}_{app}$. Figure 3(a) shows that the effective FE capacitance, ${C}_{fe}^{ef{f-awg}}=dQ/d{V}_{fe}^{avg}$ is indeed negative while FE is in MD state which implies that the effective average permittivity of the FE layer in the out-of-plane direction, ${\varepsilon }_{z,fe}^{eff-avg}$ is negative.

The DW-motion induced negative effective permittivity can be described as follows. When ${V}_{app}$ = 0 V, the P$\downarrow$ and P$\uparrow$ domains in FE are equal in size and the local ${E}_{z,fe}$ (depolarizing field) is directed opposite to the local $P$ (i.e. P$\downarrow$ domains exhibit E$\uparrow$ and P$\uparrow$ domains exhibit E$\downarrow$). Note that ${f}_{x,grad}$ is non-zero inside of the domain (due to DW diffusion in soft-DW) and that causes the $P$ to decrease in magnitude (discussed earlier). Now, with the increase in ${V}_{app}$, P$\downarrow$ domains grow and P$\uparrow$ domains shrink in size, due to positive stiffness of DW motion^8^. As the DW moves away from P$\downarrow$ domain and towards the P$\uparrow$ domain, ${f}_{x,grad}$ in P$\downarrow$ domain decreases and in P$\uparrow$ domain increases. Due to this effect as well as increase in ${V}_{app}$, the magnitude of local $P$ in P$\downarrow$ domain increases and in P$\uparrow$ domain decreases. Our simulation shows that as a result of this, the depolarizing field (${E}_{z,fe}$) in P$\downarrow$ domain increases and in P$\uparrow$ domain decreases in magnitude. This implies ${f}_{dep}$ increases (decreases) in P$\downarrow$ (P$\uparrow$) domain. The increase (decreases) in ${f}_{dep}$ in P$\downarrow$ (P$\uparrow$) domains is possible as it is accompanied by a decrease (increase) in ${f}_{x,grad}$. As the oppositely directed local E-field in FE increases (decreases) with the increase (decrease) in local $P$ in both P$\downarrow$ and P$\uparrow$ domains, the effective local permittivity of the domains (${\varepsilon }_{z,fe}^{eff}$) become negative. At the same time, in the DW, the asymmetry in $P$ distribution (due to unequal P$\uparrow$ and P$\downarrow$ domain sizes and $P$ magnitudes) causes ${F}_{dw}$ (comprised of ${f}_{x,grad}$ and ${f}_{x,elec}$) to decrease compared to the symmetric $P$ distribution^2^ (at *V*_*app*_ = 0 V). Such a decrease in ${F}_{dw}$ allows a further increase in average ${E}_{z,fe}^{avg}$ (an increase in depolarization energy) in the DW, while the average-$P$ (directed opposite to ${E}_{z,fe}^{avg}$) in the DW increases (due to unequal $P$ magnitudes in P$\uparrow$ and P$\downarrow$ domain). As a consequence, the permittivity of the DW region also becomes negative. These effective local (and non-homogeneous) negative permittivity (${\varepsilon }_{z,fe}^{eff}$) of the domain and DW regions give rise to an average effective negative permittivity in the FE layer, i.e. ${\varepsilon }_{z,fe}^{eff-avg} < 0$.

Here, it is important to note that the appearance of negative effective permittivity is essentially an apparent phenomena of change in long range interaction of $P$, its gradient and/or DW energy under DW motion. In particular, the change in $P$ in MFIM is not directly driven by the local E-field, rather, the change in $P$ is driven by the applied E-field induced domain-wall motion. Therefore, the change in local E-field is the effect of change in $P$ and not the opposite. In other words, the depolarizing E-field appears depending on the change in $P$ induced by DW motion. Even though, such phenomena leads to a negative effective permittivity of the FE layer, the susceptibility of the FE layer and the whole system (MFIM stack) is positive with respect to the applied E-field. That implies, the change in polarization is always in the direction of the change in applied E-field.

As we have identified that the ${F}_{dw}$ plays a crucial role in providing negative ${\varepsilon }_{z,fe}^{eff-avg}$, therefore, it is intuitive that the NC effect is dependent on its components, i.e. ${f}_{x,grad}$ and ${f}_{x,elec}$. To investigate such dependency, the average effective NC path in the $Q$-${E}_{z,fe}^{avg}$ responses of MFIM stack for different $g$ are shown in Fig. 3(c). Here, ${E}_{z,fe}^{avg}$ is the z-component of E-field in FE averaged along the length (x-axis), which we calculate as ${E}_{z,fe}^{avg}={V}_{fe}^{avg}/{T}_{fe}$. Figure 3(c) shows an increase in the NC effect with an increase in $g$. Here, similar to the earlier works^2–4^, an increase in the NC effect implies an increase in $1/|{\varepsilon }_{z,fe}^{eff-avg}|=|d{E}_{z,fe}^{avg}/dQ|$. As the ${f}_{x,grad}$ increases with the increase in $g$, a higher energy modulation is achieved by displacing the DW, which further provides a higher increase (or decrease) in ${f}_{dep}$ in P$\downarrow$ (or P$\uparrow$) domains, leading to larger NC effect. Similarly, $dP/dx$ increases as the number of domains and the DWs increase with the decrease in ${T}_{fe}$ (discussed before). Therefore, ${f}_{x,grad}$ increases and provides an increased NC effect with decreasing ${T}_{fe}$ (Fig. 3(d)). However, the soft-DW induced NC path does not depend of ${T}_{de}$ (Fig. 3(e)). This is because, in the MD state, the average depolarization field (which is zero at *V*_*app*_ = 0) as well as ${f}_{x,grad}$ and ${f}_{x,elec}$ are independent of ${T}_{de}$ within the limit of soft-DW. Interestingly, the MD-NC path does depend on the relative DE permittivity (${\varepsilon }_{de}$) as shown in Fig. 3(f). This is because the in-plane E-field, ${E}_{x,fe}$ in the DW needs to satisfy the in-plane boundary condition at the FE-DE interface, which is ${E}_{x,fe}={E}_{x,de}$, where ${E}_{x,de}$ and ${E}_{x,fe}$ are the in-plane E-field in DE and FE, respectively. As the ${E}_{x,de}$ increases with the decrease in ${\varepsilon }_{de}$ (considering similar $P$ difference between two consecutive domains), therefore, ${E}_{x,fe}$ also increases in FE, which further increases the ${f}_{x,elec}$ stored in the DW. Therefore, the ${F}_{dw}$ increases and hence, NC effect increases with the decrease in ${\varepsilon }_{de}$ as shown in Fig. 3(f). From this analysis, we can summarize that, (i) an FE material with higher $g$, (ii) ${T}_{fe}$ scaling and/or (iii) using DE materials with low ${\varepsilon }_{de}$ are key design knobs to enhance DW-induced NC effect in MFIM stack. Note that in all of the cases discussed above, the MD NC path does not coincide with the Landau path (Fig. 3(c–f)) and the MD NC effect is less ($\mathrm{1/|}{\varepsilon }_{z,fe}^{eff-avg}|$ is less) compared to the NC effect that corresponds to Landau path.

**Figure 4:**
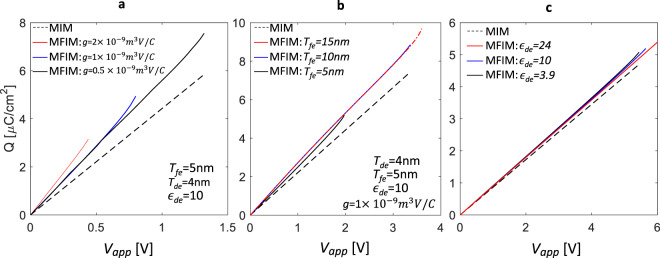
*Q*-*V*_*app*_ response of MIM and MFIM for different (**a**) *g*, (**b**) *T*_*fe*_, and (**c**) *ε*_*de*_ (but same *C*_*MIM*_ = *ε*_0_*ε*_*de*_/*T*_*de*_) for the range of |*V*_*app*_| within which the MD state is retained. The lateral dimension (along the *x*-axis) of 100 nm is used for all simulations.

As the MD NC path is dependent on ${T}_{fe}$, $g$ and ${\varepsilon }_{de}$, therefore, the charge enhancement characteristics also depend on them as shown in Fig. 4(a–c). For simplicity, we only show the charge response for ${V}_{app} > 0$. Now, a relation between the charge response in MFIM (${Q}_{MFIM}$) and MIM (${Q}_{MIM}$) can be written as the following equation.

3 $$\frac{{dQ}_{MFIM}}{{dQ}_{MIM}}=\frac{{dQ}_{MFIM}/{dV}_{app}}{{dQ}_{MIM}/{dV}_{app}}={\left(1-\frac{{C}_{MIM}\times {T}_{fe}}{|{\varepsilon }_{z,fe}^{eff-avg}|}\right)}^{-1}$$

Here ${C}_{MIM}$ (=${\varepsilon }_{0}{\varepsilon }_{de}/{T}_{de}$) is the MIM capacitance per unit area. Now, considering that an increase(decrease) in charge enhancement implies an increase(decrease) in the right-hand side of the above equation, let us discuss the $g$, ${T}_{fe}$, and ${\varepsilon }_{de}$ dependency. The charge enhancement in MFIM increases with the increase in $g$ (as $\mathrm{1/|}{\varepsilon }_{z,fe}^{eff-avg}|$ increases) as shown in Fig. 4(a). Now with the increase in ${T}_{fe}$, $\mathrm{1/|}{\varepsilon }_{z,fe}^{eff-avg}|$ decreases. However, increase in ${T}_{fe}$ dominates over decrease in $\mathrm{1/|}{\varepsilon }_{z,fe}^{eff-avg}|$, when ${T}_{fe}$ increases from 5 nm to 10 nm and therefore, charge enhancement shows mild increase (due to counteracting factors) as shown in Fig. 4(b). In contrast, when ${T}_{fe}$ increases from 10 nm to 15 nm, the increase in ${T}_{fe}$ is almost compensated by decrease in $\mathrm{1/|}{\varepsilon }_{z,fe}^{eff-avg}|$ and hence, charge enhancement shows almost similar characteristics (Fig. 4(b) for *T*_*fe*_ = 10 nm and 15 nm). Now, with the increase in ${\varepsilon }_{de}$ (but same ${C}_{MIM}$, obtained by increasing ${T}_{de}$ proportionately, so the $Q$-${V}_{app}$ characteristics of MIM remains same), charge enhancement increases as shown in Fig. 4(c). This is because, $\mathrm{1/|}{\varepsilon }_{z,fe}^{eff-avg}|$ increases with the decrease in ${\varepsilon }_{de}$.

Based on the above discussion, we emphasize that the negative effective permittivity of the MD state is not an intrinsic material parameter of FE, rather, it depends on the physical thickness of the FE film, its gradient energy coefficient and the permittivity of the underlying DE material. It is important to note that, this conclusion is different than a single domain model^1^, where the negative permittivity originates from negative slope of the Landau-Khalatnikov (LK) equation and hence, remains constant irrespective of ${T}_{fe}$ and ${\varepsilon }_{de}$. Further, ${\varepsilon }_{z,fe}^{eff-avg}$ is a non-local quantity and can only describe the average characteristics. Moreover, the value of ${\varepsilon }_{z,fe}^{eff-avg}$ obtained for a particular ${T}_{fe}$ and ${\varepsilon }_{de}$ can not be used to calculate the average charge response of MFIM/MFIS stack with any other ${T}_{fe}$ and ${\varepsilon }_{de}$ due to the dependency of ${\varepsilon }_{z,fe}^{eff-avg}$ on these parameters. However, ${\varepsilon }_{z,fe}^{eff-avg}$ can be used to calculate the average charge response of MFIM/MFIS stack with different ${T}_{de}$ within the limit of soft DW formation. Furthermore, the local effective permittivity, ${\varepsilon }_{z,fe}^{eff}$ of is not an intrinsic material property and hence, spatially varies within the FE layer^3,4^. As the DW moves with the applied voltage, the local effective permittivity value changes with ${V}_{app}$. As, ${\varepsilon }_{z,fe}^{eff}$ is not a spatially static quantity, therefore, one cannot directly use this in a capacitor equation to analyze the local charge response of the heterostructures (i.e. MFIM and MFIS stack). However, ${\varepsilon }_{z,fe}^{eff-avg}$ can be used in a capacitor equation to calculate the average charge response of MFIM/MFIS stack (within the limit of MD state) as in Eqs. 3–4. Therefore, the reason for introducing ${\varepsilon }_{z,fe}^{eff}$ and ${\varepsilon }_{z,fe}^{eff-avg}$ in this work is to analyze the implication of different parameters and to emphasize that, one should not use this characteristics as an intrinsic property of FE.

## MD-NC Effect in MFIS Stack

**Figure 5:**
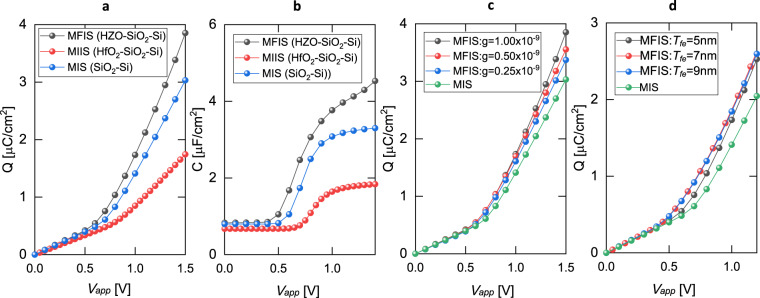
(**a**) *Q*-*V*_*app*_ and (**b**) *C*-*V*_*app*_ of MFIS (HZO(5 nm)-SiO_2_(1 nm)-Si(10 nm)), MIS (SiO_2_(1 nm)-Si(10 nm)), MIIS (HfO_2_(5 nm)-SiO_2_(1 nm)-Si(10 nm)) stacks. Here, $g=1\times {10}^{-9}\,{m}^{3}V/C$. *Q*-*V*_*app*_ of MFIS stack for different (**c**) *g* and (**d**) *T*_*fe*_ and their comparison with MIS stack. The lateral dimension (along the *x*-axis) of 100 nm is used for all simulations.

So far, we discussed how the DW-induced effective NC in FE can enhance the overall charge response of MFIM stack. Next, we turn our attention to the MFIS stack with an undoped silicon as the semiconductor layer. To compare the MFIS results with conventional MOS capacitor, we also simulate MIIS (Metal-HfO_2_-SiO_2_-Si) and MIS (Metal-SiO_2_-Si) stacks. The silicon layer thickness of 10 nm is considered for all the simulations of MFIS, MIIS, and MIS stacks. The simulated $Q$-${V}_{app}$ and $C$-${V}_{app}$ (capacitance, $C=dQ/d{V}_{app}$) responses are shown in Fig. 5(a,b), which illustrate an enhanced charge and capacitance response of MFIS compared to the MIIS and MIS stacks. We attribute this enhanced charge/capacitance response to the negative ${\varepsilon }_{z,fe}^{eff-avg}$ of FE that we discussed earlier for MFIM. Now, to analyze the effects of ${T}_{fe}$, $Q$-${V}_{app}$ characteristics for different ${T}_{fe}$ are illustrated in Fig. 5(d) showing minor enhancement in the charge response with an increase in ${T}_{fe}$. To understand this, a relation can be derived between the charge response in MFIS (${Q}_{MFIS}$) and in MIS (${Q}_{MIS}$) when ${\varepsilon }_{z,fe}^{eff-avg}$ is negative as follows.

4 $$\frac{{dQ}_{MFIS}}{{dQ}_{MIS}}=\frac{{dQ}_{MFIS}/{dV}_{app}}{{dQ}_{MIS}/{dV}_{app}}={\left(1-\frac{{C}_{MIS}\times {T}_{fe}}{|{\varepsilon }_{z,fe}^{eff-avg}|}\right)}^{-1}$$

Here, ${C}_{MIS}$ is the capacitance per unit area of MIS stack. Recall that, $\mathrm{1/|}{\varepsilon }_{z,fe}^{eff-avg}|$ decreases with the increase in ${T}_{fe}$ (discussed for MFIM). However, the increase in ${T}_{fe}$ dominates over decrease in $\mathrm{1/|}{\varepsilon }_{z,fe}^{eff-avg}|$ in the expression of ${Q}_{MFIS}$ above. Consequently, the charge responses show a mild boost (1.01x) with the increase in ${T}_{fe}$ (due to two counteracting factors). Similarly, to analyze the effect of ${f}_{x,grad}$, we simulate MFIS stack for different values of $g$. The $Q$-${V}_{app}$ characteristics (Fig. 5(c)) show that the MFIS charge response enhances with the increase in $g$ and are higher than the corresponding MIIS and MIS stacks. This is because the NC effect enhances ($\mathrm{1/|}{\varepsilon }_{z,fe}^{eff-avg}|$ increases) with the increase in $g$, as we discussed earlier in the context of MFIM.

**Figure 6:**
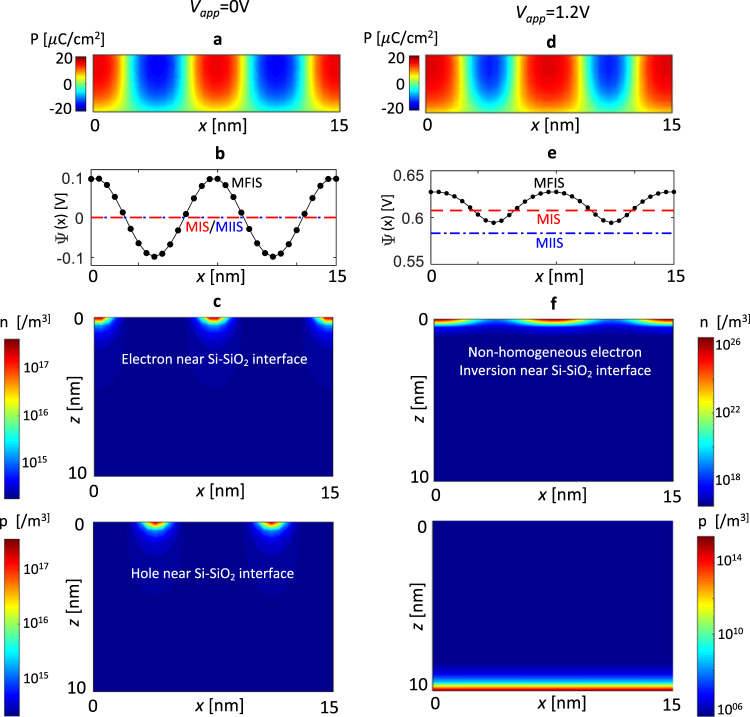
(**a**) $P(x,z)$ of FE, (**b**) surface potential (Ψ(x)), and (**c**) electron concentration (*n*) and hole concentration (*p*) in Si layer of MFIS stack at *V*_*app*_ = 0 V. (**d**) $P(x,z)$ of FE, (**e**) Ψ(*x*), and (**f**) $n$ and $p$ in Si layer of MFIS stack at *V*_*app*_ = 1.2 V. Here, *T*_*fe*_ = 5 nm, *T*_*de*_ = 1 nm (SiO_2_), *T*_*si*_ = 10 nm and $g=1\times {10}^{-9}\,{m}^{3}V/C$. The lateral dimension (along the *x*-axis) of 100 nm is used for all simulations.

**Figure 7:**
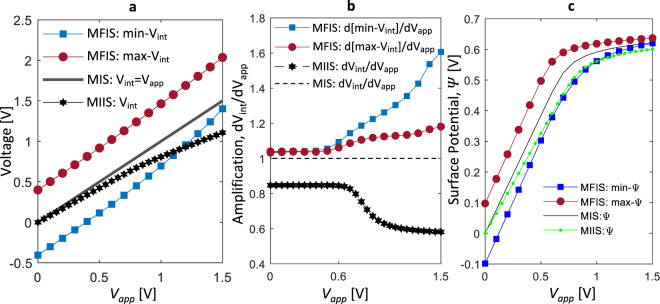
(**a**) FE-DE interface potential, *V*_*int*_ of MFIS, MIS and MIIS stacks. (**b**) d*V*_*int*_/d*V*_*app*_ of MFIS, MIS and MIIS stacks showing differential amplification of min/max-*V*_*int*_ in MFIS stack. (**c**) Surface potential, Ψ of MFIS, MIS and MIIS stacks. Here, *T*_*fe*_ = 5 nm, *T*_*de*_ = 1 nm (SiO_2_), *T*_*Si*_ = 10 nm and $g=1\times {10}^{-9}\,{m}^{3}V/C$. The lateral dimension (along the *x*-axis) of 100 nm is used for all simulations.

The overall enhancement in charge/capacitance response of MFIS stack (compared to MIS and MIIS) can be easily understood from the effective negative ${\varepsilon }_{z,fe}^{eff-avg}$ of FE. However, for FE-FET operation, it is also important to analyze the semiconductor surface potential (Ψ) in MFIS, especially, when FE is in MD state (Fig. 6(a)). In fact, Ψ(*x*) in MFIS is non-homogeneous as shown in Fig. 6(b) at *V*_*app*_ = 0 V. To understand this, let us consider the potential at the FE-DE interface, ${V}_{int}(x)$. Note that in the MD state, E-field in FE, ${E}_{z,fe}$ (=$({V}_{app}-{V}_{{int}}(x))/{T}_{fe}$) is directed opposite to the local $P$ and exhibits a non-homogeneous profile along the x-direction due to periodic P$\uparrow$ and P$\downarrow$ domains. Therefore, ${V}_{int}(x)$ becomes non-homogenous and exhibits a maxima (max-${V}_{int}$) and minima (min-${V}_{int}$) corresponding to the center of P$\downarrow$ and P$\uparrow$ domains, respectively. This non-homogeneity in ${V}_{int}(x)$ induces a spatially varying Ψ(*x*) as shown in Fig. 6(b), which, in turn exhibits a maxima (max-Ψ) and minima (min-Ψ). This further leads to local accumulation and co-existence of electrons or holes in the undoped Si layer (Fig. 6(c)). Note, such a spatially varying charge profile has been experimentally shown in ref. ^16^ for FE-semiconductor interface, when FE is in MD state. Now, with the increase in ${V}_{app}$ (~1.2 V), P$\downarrow$ domains grow and P$\uparrow$ domains shrink in size leading to an overall increase in average $P$ (Fig. 6(d)). Simultaneously, min/max-${V}_{int}$ increases (Fig. 7(a)) and at the same time exhibits a differential amplification ($d{V}_{int}/d{V}_{app} > 1$) as shown in Fig. 7(b). Here the local differential amplification in min/max-${V}_{int}$ can be attributed to the effective local negative permittivity of FE in the P$\downarrow$ and P$\uparrow$ domains (discussed for MFIM). Now, as ${V}_{int}$ increases, Ψ everywhere at the Si interface increases and becomes positive (but still remains non-homogeneous, see Fig. 6(e)). Therefore, electron density ($n$) dominates over hole density ($p$) locally and globally (Fig. 6(f)). Note that the increase in $n$ causes the non-homogeneity in Ψ to decrease (Fig. 6(e,f)) compared to *V*_*app*_ = 0 V (Fig. 6(b,c)). The Ψ for MFIS, MIIS and MIS stacks for ${V}_{app}$ = 0 V and 1.2 V are shown in Fig. 6(b,e). At ${V}_{app}=0\,V$, the max(min)-Ψ in MFIS is higher(lower) than the MIIS and MIS stacks. At ${V}_{app}$ = 1.2 V, the max-Ψ in the MFIS is higher than the MIIS and MIS and the min-Ψ in MFIS is higher than MIIS but lower than the MIS. This can be understood from the following discussion. As in the MD state, ${E}_{z,fe}$ (=$({V}_{app}-{V}_{int}(x))/{T}_{fe}$) is directed opposite to the local $P$, therefore, the max-${V}_{int}$ is larger than ${V}_{app}$ (for P$\downarrow$ domains with E$\uparrow$ i.e. ${E}_{z,fe} < 0$) and the min-${V}_{int}$ remains less than ${V}_{app}$ (for P$\uparrow$ with E$\downarrow$ i.e. ${E}_{z,fe} > 0$). This holds true when the FE is in 180° MD state and an only exception to this (where min-${V}_{int} > {V}_{app}$ can occur) is for a very small voltage window just before the MD state switches to the poled state (discussed in the Supplementary Section for MFIM). Hence, as long as FE remains in the 180° MD state (i.e. does not switch to the poled state), the min(max)-${V}_{int}$ is always lower(higher) than ${V}_{app}$ in MFIS (see Fig. 7(a)). Note that, this statement is also true for MFIM and is discussed in the Supplementary Section. Now, in the MIS stack, DE potential is directly driven by ${V}_{app}$ and hence ${V}_{int}={V}_{app}$. Therefore, min-${V}_{int}$ of MFIS is always less than ${V}_{int}$ (=${V}_{app}$) of MIS as shown in Fig. 7(a). In addition, *d*Ψ/*dV*_*int*_ is <1 and equal for both MFIS and MIS due to the same positive capacitance of the DE layer. As a consequence, the min-Ψ of MFIS is inevitably lower than the Ψ of MIS, when the FE is in 180° MD state (Fig. 7(c)). However, in MIIS, the ${V}_{int}$ (HfO_2_-SiO_2_ interface potential) is not directly driven by ${V}_{app}$ and due to the positive capacitance of the HfO_2_ layer, $d{V}_{int}/d{V}_{app} < 1$ and ${V}_{int} < {V}_{app}$ (Fig. 7(b,c)). Now, considering the differential amplification of min-${V}_{int}$ in MFIS ($d(min-{V}_{int})/d{V}_{app} > 1$) as shown in Fig. 7(b), the min-${V}_{int}$ of MFIS becomes higher than the ${V}_{int}$ of MIIS beyond a certain ${V}_{app}$ (Fig. 7(c)). As a result, min-Ψ of MFIS becomes higher than the Ψ of MIIS at ${V}_{app} > 1\,\text{V}$) as shown in Fig. 7(c). Briefly, in MFIS, the min-Ψ can exceed the Ψ in MIIS but remains lower than the MIS, while the max-Ψ is always higher than the Ψ in MIIS and MIS.

Now, let us make a rough assumption that the channel current in FEFET will be mostly dependent on the min-Ψ as that determines the highest potential barrier seen by the source electrons. Then, based on the above discussion, we can expect that the OFF current (at *V*_*app*_ = 0 V) of FEFET will be significantly less compared to the MIS/MIIS-FET, and the ON current (*V*_*app*_ ~ 1.2 V) will be higher than the MIIS-FET but comparable to MIS-FET. As the Ψ is highly non-homogeneous in MFIS stack in the low voltage regime, calculation of SS of FEFETs needs further exploration by considering source/drain regions along with the DW-induced non-homogeneous semiconductor potential and solving the transport equations to obtain the impact of the MD FE on the FEFET characteristics.

## Conclusion

In summary, by performing phase-field simulation, we show that the energy stored in FE DW can be harnessed to enhance the capacitance of the MFIM and MFIS stack, where the soft-DW displacement leads to a static and hysteresis-free negative capacitance in the MD FE. Our analysis indicates that the effective negative permittivity of the FE layer is dependent on the FE thickness, gradient energy coefficient, in-plane permittivity of the DE and is independent of DE thickness within the limit of soft-DW. Further, the DW-induced NC effect can lead to an enhanced charge/capacitance response in MFIS stack compared to MIS/MIIS stack. However, such a charge/capacitance enhancement in MFIS does not guarantee an enhanced local Ψ in Si compared to MIS. In fact, Ψ becomes spatially varying due to the MD nature of FE and the variation is higher at low applied voltages. In addition, we discuss that the minimum Ψ in MFIS can exceed the Ψ in MIIS but remains smaller than the MIS. Nevertheless, considering the local differential amplification of ${V}_{int}$ (i.e. $d(min-{V}_{int})/d{V}_{app} > 1$), the on/off current ratio of FEFET can potentially exceed the MIS/MIIS-FET. Since the non-homogeneity in Ψ is absent in conventional MOS capacitor (and MOSFET), therefore, as future work, it will be important to investigate the impact of such potential profile on the low voltage conduction of FEFETs.

## Methods

In our phase-field simulation framework, we self-consistently solve the time-dependent Ginzburg-Landau (TDGL) equation, Poisson’s equation and semiconductor charge equations. Considering polarization ($P$) as the state variable, the total energy density of FE (${f}_{tot}$) can be written as following.

5 $$\begin{array}{rcl}{f}_{tot}(x,z) & = & {f}_{free}(x,z)+{f}_{grad}(x,z)+{f}_{dep}(x,z)\\ {f}_{free}(x,z) & = & {f}_{0}+\frac{\alpha }{2}P{(x,z)}^{2}+\frac{\beta }{4}P{(x,z)}^{4}+\frac{\gamma }{6}P{(x,z)}^{6}\\ {f}_{grad}(x,z) & = & \frac{{g}_{11}}{2}{\left(\frac{\partial P(x,z)}{\partial z}\right)}^{2}+\frac{{g}_{44}}{2}{\left(\frac{\partial P(x,z)}{\partial x}\right)}^{2}\\ {f}_{dep}(x,z) & = & -{E}_{{z,fe}}(x,z)\times P(x,z)\end{array}$$

Here, ${f}_{free}$ is the free energy density, ${f}_{grad}$ is the gradient energy density and ${f}_{dep}$ is the depolarization energy density. Additionally, *α*, *β* and *γ* are Landau’s free energy coefficients; ${g}_{11}$ and ${g}_{44}$ are gradient energy coefficients; and ${E}_{z},{f}_{e}$ is the E-field in FE parallel to the $P$ direction. For simplicity, we assume *g*_11_ = *g*_44_ = *g*. Now, the TDGL equation (Eq. 6) in Euler-Lagrange form (Eq. 7) can be written as following.

6 $$-\frac{1}{\varGamma }\times \frac{\partial P}{\partial t}=\frac{\delta F}{\delta P};\,here,\,F=\iint \,f(x,z)dxdz$$

7 $$\begin{array}{rcl}-\frac{1}{\varGamma }\times \frac{\partial P(x,z)}{\partial t} & = & \alpha P(x,z)+\beta {P}^{3}(x,z)+\gamma {P}^{5}(x,z)\\  &  & -\,{g}_{11}\frac{{\partial }^{2}P(x,z)}{\partial {z}^{2}}-{g}_{44}\frac{{\partial }^{2}(x,z)P}{\partial {x}^{2}}-{E}_{z},{f}_{e}(x,z)\end{array}$$

Here, $z$ and $x$ are the axis along the thickness and length of the stack, respectively. Eq. 7 is also known as Chensky-Tarasenko (CT) equation, and has been used to analyze polarization switching characteristics in DE-FE-DE stack^10^. Note that, here we do not include the strain as a separate order parameter (which has been done for standalone FE in earlier works^17^). Rather, by considering strain-polarization coupling and assuming a stress free condition a similar equation as Eq. 7 can be derived, where the Landau coefficients incorporates both of the strain and polarization contribution of the free energy density (see Supplementary Section of ref. ^14^). Now, for the considered system, the Poisson’s equation can be written as,

8 $$\begin{array}{c}-{\varepsilon }_{0}\left[\frac{\partial }{\partial x}\left({\varepsilon }_{x}(x,z)\frac{\partial \varPhi (x,z)}{\partial x}\right)+\frac{\partial }{\partial z}\left({\varepsilon }_{z}(x,z)\frac{\partial \varPhi (x,z)}{\partial z}\right)\right]\\ \,=\,\frac{\partial P(x,z)}{\partial z}-\rho (x,z)\end{array}$$

Here, Φ$(x,z)$ is the potential, ${\varepsilon }_{x(z)}$ is the relative permittivity of the material along the x(z)-direction and $\rho (x,z)$ is the charge density. Note, $\rho (x,z)\ne 0$ only in the semiconductor region and has been calculated from the following equations.

9 $$\begin{array}{rcl}\rho (x,z) & = & q(p(x,z)-n(x,z)-{N}_{a}^{-}(x,z)+{N}_{d}^{+}(x,z))\\ n(x,z) & = & {\int }_{{E}_{c}}^{\infty }\,\frac{8\pi \sqrt{2}}{{h}^{3}}{m}_{e}^{\ast 3/2}\sqrt{E-{E}_{c}}[{f}_{f-d}(E-q\varPhi (x,z))]dE\\ p(x,z) & = & {\int }_{-\infty }^{{E}_{v}}\,\frac{8\pi \sqrt{2}}{{h}^{3}}{m}_{h}^{\ast 3/2}\sqrt{{E}_{v}-E}[1-{f}_{f-d}(E-q\varPhi (x,z))]dE\end{array}$$

Here, ${f}_{f-d}$ is the fermi-dirac distribution function. ${N}_{a}^{-}$ and ${N}_{d}^{+}$ are the concentration of ionized acceptor and donor, respectively, which are negligible in our case due to the consideration of undoped silicon as the semiconductor layer in the MFIS stack. Now, for MFIM stack, the self-consistent solution for $P(x,z)$ and Φ(*x*, *z*) are obtained by solving Eqs. 7 and 8. For MFIS stack, the self-consistent solution for $P(x,z)$, $\rho (x,z)$ and Φ(*x*, *z*) are obtained using Eqs. 7–9. Note that the solution of the Poisson’s equation provides potential profile (Φ(*x*, *z*)) and E-field profile (${E}_{x}(x,z)=-\,d$Φ(*x*, *z*)/*dx*, ${E}_{z}(x,z)=-\,d$Φ(*x*, *z*)/*dz* of the system for a given polarization profile of FE ($P(x,z)$) and charge profile of Si ($\rho (x,z)$). Then we use the ${E}_{{z,fe}}(x,z)$ in TDGL equation (Eq. 7) to calculate the $P(x,z)$ and Φ(*x*, *z*) in the semiconductor-charge equation (Eq. 9) to calculate the $\rho (x,z)$. We solve these equations (Eqs. 7–9) in real space by employing finite difference method with grid spacing of Δ*x* = Δ*z* = 0.5 nm by considering Dirichlet boundary condition at the top and bottom metal contacts (i.e. by defining the voltage) and periodic boundary condition at the left and right edges of the system. For all the simulation results presented in this work, the lateral (along $x$-axis) dimension of the system is considered as 100 nm.

The Landau’s free energy coefficients (*α*, *β* and *γ*) of Hf_0.5_Zr_0.5_O_2_ (HZO) are extracted from measured *P*-*V* characteristics of 10 nm HZO film^12^. The experimental *P*-*V* characteristics and ‘Landau path’ are shown in Fig. 1(c). Here the ‘Landau path’ implies the solution of Eq. 10, which can be written from Eq. 7 by assuming static scenario and ideal metal-ferroelectric-metal capacitor (so that, at poled state, $dP(x,z)/dx=dP(x,z)/dz=0$).

10 $$V/{T}_{FE}=\alpha P+\beta {P}^{3}+\gamma {P}^{5}$$

All the simulation parameters are listed in Table 1. Here, the rest mass of electron, *m*_0_ = 9.11 × 10^-31^ kg and the vacuum permittivity, *ε*_0_ = 8.854 × 10^-12^ F/m.

**Table 1:** Simulation parameters.

Parameter	Value	Parameter	Value
*α*	$-\,2.5\times {10}^{9}\,Vm/C$	*ε*_*fe*_	24 (HZO)
*β*	$6.0\times {10}^{10}\,V{m}^{5}/{C}^{3}$	*ε*_*Si*_	11.7
*γ*	$1.5\times {10}^{11}\,V{m}^{9}/{C}^{5}$	${m}_{e}^{\ast }$	1.08 × *m*_0_
*g* (=*g*_11_ = *g*_44_)	$2\times {10}^{-10}\,V{m}^{3}/C$	${m}_{h}^{\ast }$	0.81 × *m*_0_
	$1\times {10}^{-10}\,V{m}^{3}/C$	*ε*_*de*_	3.9 (SiO_2_)
	$0.5\times {10}^{-10}\,V{m}^{3}/C$		10 (Al_2_O_3_)
	$0.25\times {10}^{-10}\,V{m}^{3}/C$		24 (HfO_2_)

## Supplementary information

Supplementary Information.
