# Supplementary material for: Multi-Domain Negative Capacitance Effects in Metal-Ferroelectric-Insulator-Semiconductor/Metal Stacks: A Phase-field Simulation Based Study
Source: Sci Rep. 2020 Jun 23;10:10207. doi: 10.1038/s41598-020-66313-1 (PMC7311392; doi:10.1038/s41598-020-66313-1)
Supplement: Supplementary file 1 — Supplementary Information. [file 41598_2020_66313_MOESM1_ESM.pdf]

<u>Supplementary Section</u>

Multi-Domain Negative Capacitance Effects in Metal-Ferroelectric-Insulator-Semiconductor/Metal Stacks: A Phase-field Simulation Based Study

Atanu K Saha[1,*] and Sumeet K Gupta[1]

[1]School of Electrical and Computer Engineering, Purdue University, West Lafayette, IN, 47906, USA

*saha26@purdue.edu

Multi-domain (MD) formation in MFIM from poled state:

In the main text we discussed that the Q-V_{APP} characteristics can become hysteretic if the formation of the MD state occurs from the poled state. The simulated Q-V_{APP} characteristics is shown in Fig. S1(a) for (T_{FE}=5nm, T_{DE}=4nm (Al_2O_3), g=1x10^{-9}m^{3}V/C). At (i) V_{APP}=0V and the FE is in MD state (P↑↓). From (i) to (iii), average P increases via DW motion (increase in P↓ domain and decrease in P↑ domain size) with the increase in V_{APP}. Now, with the further in V_{APP}, the FE undergoes a transition from MD to poled state with P↓ (at |V_{APP}|=2V, from (iii) to (iv)). Now, with decreasing |V_{APP}|, MD state forms from the poled state at a lower |V_{APP}| (~0.9V) and that induces a hysteresis in the Q-V_{APP} characteristics. It is important to note that the physical mechanism for applied voltage dependent transition from a poled state to 180° MD state depends on several factors. In an ideal scenario, such a transition occurs only after crossing the intrinsic (or microscopic) nucleation barrier (Landau's free energy barrier). In contrast, the presence of local nucleation sites, grain boundaries, defects, disorders and other non-ideal factors can trigger an early nucleation in FE materials, which has been extensively investigated and still under investigation to resolve the famous 'Landauer's Paradox' of domain nucleation [S1-S2]. Therefore, for an exact estimation of hysteresis due to the formation of MD state from poled state, further studies are required by considering finite size effects of FE grains and other non-ideal factors in the phase field simulation.

Non-homogeneous V_{INT} in MFIM:

When FE is in MD state, E-field in FE, $E_{FE,Z}$ (≈(V_{APP}-V_{INT})/T_{FE}) is directed opposite to the local P and exhibits a spatially non-homogeneous profile along x-direction due to the periodic P↑ and P↓ domains. Therefore, potential at the FE-DE interface (V_{INT}) becomes non-homogeneous along the x-direction and exhibits a maxima (max-V_{INT}) and minima (min-V_{INT}) as shown in Fig. S1(b). The P-map and V_{INT} for different applied voltages (V_{APP}) are shown in Fig. S1 (c-d). As in MD state, $E_{FE,Z}$ (≈(V_{APP}-V_{INT})/T_{FE}) is directed opposite to the local P, therefore, the max-V_{INT} is higher than V_{APP} (corresponds to P↓ domain where E↑) and the min-V_{INT} remains less than V_{APP} (corresponds to P↑ where E↓). This holds true when the FE is in 180° MD state and an

only exception to this (where min-V_{INT}>V_{APP}) can happen for a very small voltage window just before the MD state switches to poled state, which can be seen in Fig. S1(c) from point (ii) to point (iii). Note that in this small voltage window, *P*-direction in P↑ domain remains ↑ inside of the domain but becomes ↓ near the DE interface. This is because the local E becomes ↑ as the min-V_{INT}>V_{APP}. Hence, the FE no longer has 180° DWs; rather, the DW tends to form along the thickness of the film. Considering the ultrathin nature of the film (~5nm), a DW along the thickness direction becomes unstable and therefore, the P↑ domain completely switches to ↓, which gives rise to homogeneously poled-P↓ state. Hence, as long as FE remains in the 180° MD state, the max(min)-V_{INT} is always higher(lower) than V_{APP}.

References:

[S1]A. Jiang, H. J. Lee, C. S. Hwang, and T. Tang, Phys. Rev. B 80, 024119 (2009).

[S2]C. Durkan, A. Hershkovitz, D. Chu, J. F. Scott, Y. Ivry, arXiv:1608.03890.

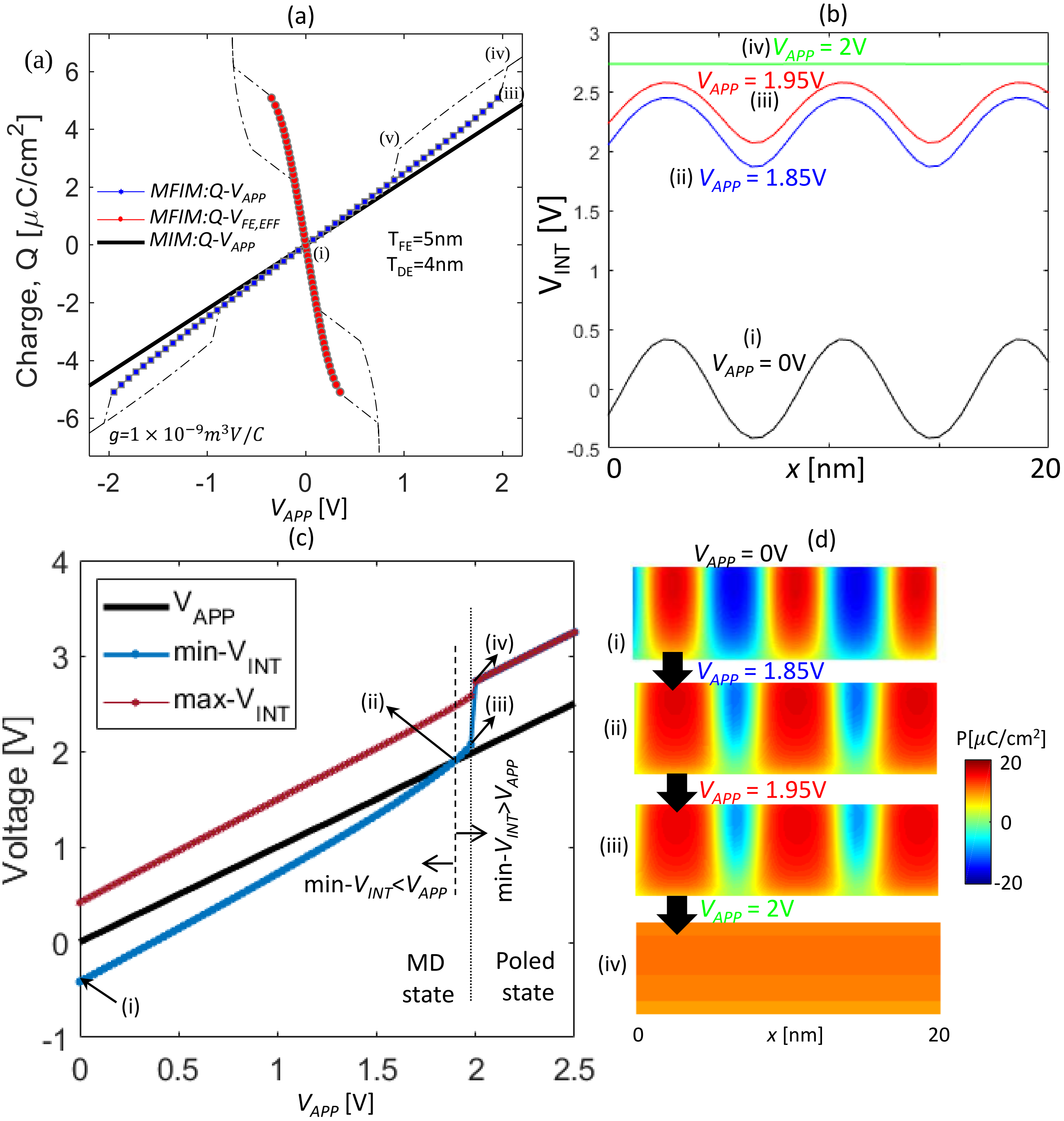

FIG. S1. (a) Q-V_{APP} characteristics of MFIM stack when FE is in MD state (blue) and MIM stack for T_{DE}=4nm. The black-dashed line represents the poled condition (if V_{APP}>2V). Extracted Q-$V_{FE,EFF}$ response (red-circle). (b) Potential at FE-DE interface (V_{INT}) at different V_{APP}. (c) max-V_{INT} and min-V_{INT} with respect to V_{APP}. (d) P-map at different V_{APP} as marked in (b,c).
